# What is the agreement between principles and practice of antibiotic stewardship in the management of diabetic foot infection: an in-hospital quality control study

**DOI:** 10.5194/jbji-9-183-2024

**Published:** 2024-06-28

**Authors:** Noémie Reinert, Katinka Wetzel, Fabian Franzeck, Mario Morgenstern, Markus Aschwanden, Thomas Wolff, Martin Clauss, Parham Sendi

**Affiliations:** 1 Center for Musculoskeletal Infections (ZMSI), Department of Orthopedic and Trauma Surgery, University Hospital Basel, Basel, 4031, Switzerland; 2 Department of Research and Analytic Services, University Hospital Basel, Basel, 4031, Switzerland; 3 Department of Angiology, University Hospital Basel, Basel, 4031, Switzerland; 4 Department of Vascular Surgery, University Hospital Basel, Basel, 4031, Switzerland; 5 Institute for Infectious Diseases, University of Bern, Bern, 3001, Switzerland

## Abstract

**Introduction**: Standardization of diagnostic and treatment concepts in diabetes-related foot infection (DFI) is challenging. In 2019, specific recommendations regarding diagnostic principles and antibiotic therapy (ABT) for DFI, including the one for osteomyelitis (DFO), were introduced in our institution. In this study, we assessed the adherence to these in-house guidelines 2 years after their implementation. **Methods**: Adult patients with DFI with and without DFO who underwent surgical intervention between 2019 and 2021 were included. Patients' charts were retrospectively reviewed. Accordance to recommendations regarding biopsy sampling, labeling, requesting microbiological and histopathological examinations, and treatment duration were assessed. **Results**: A total of 80 patients with 117 hospital episodes and 163 surgical interventions were included; 84.6 % required an amputation. Patients with HbA1c levels of <6.5 % more often required a revision during the same hospitalization than those with HbA1c levels of ≥6.5 % (29.4 % vs. 12.1 %, respectively, p=0.023 ). Specimens were obtained in 71.8 % of operations and sent for histological examination in 63.2 %. The mean duration of ABT was 9 (interquartile range (IQR) 5–15) d in macroscopically surgically cured episodes and 40.5 (IQR 15–42) d in cases with resection margins in non-healthy bone ( p<0.0001 ). Treatment duration results were similar when using histological results: 13 (IQR 8–42) d for healthy bone vs. 29 (IQR 13–42) d for resection margins consistent with osteomyelitis ( p=0.026 ). **Conclusion**: The adherence to recommendations in terms of biopsy sampling was good, moderate for histopathological analysis and poor for labeling the anatomic location. Adherence to recommendations for ABT duration was good, but further shortening of treatment duration for surgically cured cases is necessary.

## 1 Introduction

The overuse of antimicrobial agents is common in diabetes-related foot infections (DFIs). Appropriate thresholds for the duration and administration of antibiotic therapy (ABT) in DFI are still under debate, with a tendency toward shorter treatment algorithms (Gariani et al., 2019; Haug et al., 2022; Waibel et al., 2020; Motaganahalli et al., 2022). Adverse events, multidrug-resistant pathogens and healthcare costs are associated with prolonged ABT, making the monitoring of adequate antibiotic stewardship indispensable (Lipsky, 2016; Uçkay et al., 2019).

In the diabetic population, the presence of foot ulcerations is linked to a significant increase in all-cause mortality compared with diabetic populations without foot ulcerations (Saluja et al., 2020; Brownrigg et al., 2012). Because DFIs are associated with high rates of lower extremity amputations and frequent hospitalizations, a multidisciplinary team approach with centralized patient care, as well as adherence to international guidelines, is crucial to their treatment (Uçkay et al., 2015, 2016; Lipsky and Uçkay, 2021; Ertuğrul et al., 2020) and considered key to optimizing outcomes (Cortes-Penfield et al., 2023). In 2019, specific diagnostic and antibiotic treatment principles for DFI and diabetic foot osteomyelitis (DFO) were introduced in our institution, based on the guidelines of the International Working Group on the Diabetic Foot (Schaper et al., 2020) and Global Vascular guidelines (Conte et al., 2019; Uçkay et al., 2019). In patients requiring surgery, these principles include the labeling of anatomic localization, the number of obtained biopsies for microbiological and histopathological examination, and ABT duration based on the aforementioned findings. ABT should be stopped after complete resection of infected bone. In case of incomplete resection (i.e., signs of persistent osteomyelitis in situ), treatment is continued for 3 to a maximum of 6 weeks (Gariani et al., 2021).

This study aimed to investigate the degree of implementation of these recommendations, 2 years after their introduction, for hospitalized patients undergoing surgical intervention.

## 2 Methods

### 2.1 Diagnostic and ABT recommendations for DFI

The diagnostic principles – that were introduced in our institution in 2019 – are presented in Table 1. Biopsy samples should be sent simultaneously for microbiological and histopathological examinations. The latter is also necessary to assess a residual bone infection. In clinically stable patients, an antibiotic-free interval of 2 weeks should be respected prior to sampling. Correct labeling of the specimens with the anatomic localization is essential to interpret the findings and guide the postsurgical treatment plan. ABT should be stopped after complete resection of infected bone. This can be done immediately after surgery in cases in which the surgeon is certain (e.g., macroscopic assessment after amputation). In cases in which the surgeon is uncertain – based on macroscopic assessment – empiric ABT treatment is continued until histopathological results are available. In cases of incomplete resection (i.e., residual osteomyelitis after surgery), treatment is continued for 3 to a maximum of 6 weeks.

**Table 1:** Review of diagnostic and therapeutic principles for DFO.

Sampling after an antibiotic-free interval of (1 to) 2 weeks in clinically stable patients. Before, during and after surgery, evaluate and reassess whether the infection is limited to the skin and soft tissue or if it extends to the bone. Obtain and label tissue samples (i.e., bone samples, no swabs; a minimum of three and a maximum of six samples, if not otherwise justified). Labeling includes the anatomic localization. Obtain samples from healthy bone (i.e., proximal of the resection margin) to assess whether or not residual bone infection is present. Biopsy samples should be sent simultaneously for microbiology and histopathology examination. Samples should be sampled in such a way that the microbiology result can be correlated to the histopathological result for each single sample. If the surgeon is certain that resection is performed in a healthy bone area (e.g., amputation), no biopsy sampling is needed, provided that there is no concomitant skin- and soft-tissue infection that requires sampling to identify the causative microorganism. Antibiotic therapy should be stopped after complete resection of the infected bone. In cases of incomplete resection, treatment is continued for 3–6 weeks. In cases in which the DFI is limited to the skin- and soft-tissue infection (either before or after surgery), the antibiotic treatment duration should not be prolonged for more than 1 (to 2) week(s).

### 2.2 Study design

This study was a single-center, retrospective cohort study at the University Hospital Basel in Switzerland. Included in the study were hospitalized adult patients with DFI, including skin- and soft-tissue infection and osteomyelitis, undergoing surgical intervention at our center for musculoskeletal infection from January 2019 to December 2021. The DFI center consists of a multidisciplinary cooperation, including the Department of Orthopedic and Trauma Surgery, Angiology, Vascular Surgery, Infectious Disease, Microbiology, Endocrinology, Radiology and specialized wound-care nurses. In addition to clinical rounds on patient's ward, a multidisciplinary team meeting is held once a week to discuss complex cases.

### 2.3 Study population

**Figure 1:**
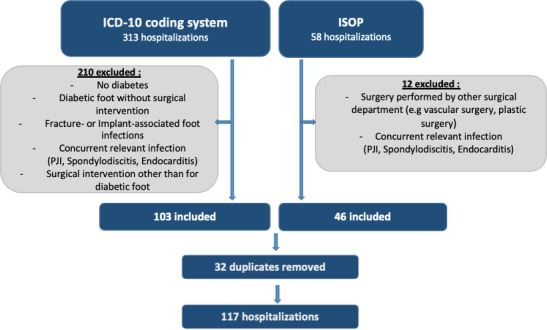
Recruitment process through the hospital intern operation program (ISOP) and ICD-10 coding system: a total of 117 hospital episodes were included after exclusion of 222 episodes.

We differentiated between the number of patients and the number of hospitalizations in the recruitment process. A hospitalization was defined as an episode with a follow-up of 12 months for revision surgery. Potentially eligible patients were identified through the in-hospital operation schedule ( n=58 ) and the ICD-10 coding system ( n=313 ). From these data sources, episodes were excluded when the patient had no diabetes mellitus or when no surgical intervention was performed. Exclusion criteria included the presence of a concomitant relevant infection that influenced ABT (e.g., infective endocarditis) or when surgery had been performed elsewhere. The flowchart of the inclusion process is illustrated in Fig. 1.

### 2.4 Variables and outcome measures

Predefined variables were retrospectively reviewed from the patient's medical records. Demographic data were assessed, including age, gender and patient-specific comorbidities. The American Society of Anesthesiologists (ASA) classification, the Charlson Comorbidity Index, the HbA1c values, and the Wound, Ischemia, and foot Infection (WIfI) score were assessed in a case-specific manner and attributed to the corresponding hospital episode. The WIfI score (Cerqueira et al., 2020) was calculated to assess the amputation risk of the lower extremity. Vascular examination and magnetic resonance imaging (MRI) findings were attributed to an episode when performed within ≤30 d prior to hospital admission. The total postoperative ABT duration was calculated from the definite (i.e., latest) surgical intervention till the last day of antibiotic prescription. Of note, in some cases, antibiotic treatment was started preoperatively, even though the patient was clinically stable. In these cases, the skin- and soft-tissue infection was moderate to severe, and there was a risk for further progression of infection if treatment was delayed. This type of decision was made by the treating team. Considering that most patients with DFI have a mixture of antibiotics and frequent changes in dosing of various agents, the following method was applied for this study: all antibiotic agents and modes of administration (parenteral, oral) were reviewed. The agent that was administered for the longest period was the one selected for data analysis of this study. The proportion of DFI treated with antibiotics according to the recommendation was the primary outcome and the proportion of antibiotic overuse the secondary outcome.

### 2.5 Statistics

Statistical analysis was performed with SPSS (Chicago, Illinois, USA). Results are presented as proportions or means with standard deviation (SD). The chi-squared test, Student's t test, Fisher's exact test and the Mann–Whitney U test were performed where appropriate. P values were considered statistically significant when they were less than 0.05 (two-sided test). The study yielded case-based results from hospital episodes.

## 3 Results

**Figure 2:**
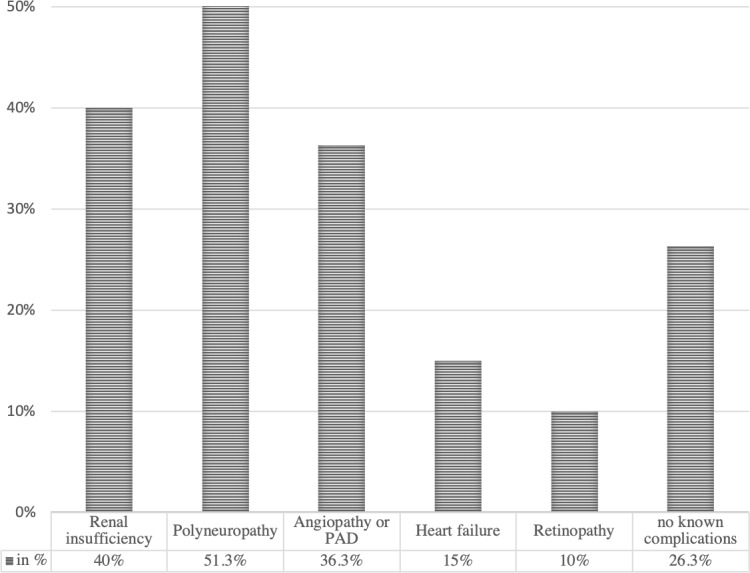
Listing of patient specific comorbidities (in %), based on a total of 80 included patients.

*Demographics and comorbidities*. A total of 80 patients with 117 hospital episodes and 163 surgical interventions were included, for a mean of 1.5 hospital episodes and 2.0 surgical interventions per patient. The mean patient age was 68.7 (SD 11.9) years, and 75 % of patients were male. Comorbidity findings per patient are demonstrated in Fig. 2. Considering that surgical and post-surgical risks for complications are associated with the ASA classification, the Charlson Comorbidity Index, the HbA1c value and the WIfI score, these results are resumed per episode in Table 2.

**Table 2:** Listing of case-based results of the Charlson Comorbidity Index and ASA classification as well as HbA1c values and WIfI score (in %), based on 117 hospital episodes.

**(a)** Charlson Comorbidity Index	%
1–2 points	8.5
3–4 points	27.4
≥5 points	64.1
**(b)** ASA classification	%
I	0.0
II	9.4
III	83.8
IV	6.8
**(c)** HbA1c values	%
Hba1c <6.5 % (prediabetes)	29.1
HbA1c 6.5 to 7.0	10.3
HbA1c 7.6 to 9.0	17.9
Hba1c >9.0	24.8
**(d)** WIfI score	%
Stage 1	3.4
Stage 2	10.3
Stage 3	30.8
Stage 4	23.9
Not available	31.6

HbA1c values demonstrated a two-sided distribution: prediabetes ( <6.5 %) was found in 29.1 % of patients and HbA1c of >9.0 % in 24.8 %. Impaired perfusion had significant differences within the HbA1c distribution ( p=0.047 ), with the highest impairment seen in HbA1c levels of 7.1 %–7.5 %, followed by HbA1c levels of 7.6 %–9.0 % (56.3 % and 53.3 %, respectively).

Vascular examination was performed in 70.9 % of episodes and MRI in 74.4 %. Impaired perfusion and DFO were confirmed by vascular examination in 34.9 % of episodes and by MRI in 56.3 %. Blood cultures were sampled in 34.2 % of patients; bacteraemia was detected in 7.7 %, with *Staphylococcus aureus* being the most common microorganism.

*Adherence to diagnostic principles*. Biopsies were obtained in 71.8 % of operations (number of biopsies: 1 in 2.4 %, 2 in 7.1 %, 3 in 77.4 %, 4 in 10.7 % and 5 in 2.4 %). For 90.5 % of these, three to five samples were taken, and 63.2 % were sent for histological examination. In 43.6 % of samples, the anatomic location was labeled “proximal to the resection margin.” Bone biopsies were collected in 94 % of cases.

*Surgical interventions*. Among all included patients, 84.6 % eventually required an amputation: 50.5 % right lower extremity with a reamputation rate of 32.7 % ( ≤12 months), 44.4 % left lower extremity with a reamputation rate of 34.7 % ( ≤12 months) and 5.1 % on both sides. Overall, 99 index amputations were recorded and 15 secondary amputations in the course of hospitalization (a total of 114 amputations). Hence, the mean number of amputations per patient was 1.4. The following heights of amputation were performed: 58.6 % toe level, 18.2 % transmetatarsal, 11.1 % forefoot or Lisfranc, 0 % Chopart, 4.0 % transtibial or Burgess, 0 % above knee and 8.1 % other levels. Among all patients included, 17.1 % needed revision surgery during the same hospital episode: 65 % one revision, 20 % two revisions and 15 % multiple revisions (>2, vascular or reconstructive surgery excluded).

*Antibiotic treatment and adherence to treatment principles*. Preoperative antibiotics were administered in 41.9 % of episodes because of concomitant skin- and soft-tissue infections. The most commonly used compound was amoxicillin–clavulanate (74.4 %). A sole intravenous administration was reported in 61.5 % of episodes. In 18.8 %, oral administration was continued after 14 d of intravenous use. In 6 %, antibiotics were only given perioperatively: 12.8 % for <1 week, 48.7 % for 1–2 weeks, 6 % for 4–6 weeks and 26.5 % for ≥6 weeks. ABT duration varied significantly when there were signs of DFO on preoperative MRI ( p=0.015 ). The mean duration of ABT was 9 (interquartile range (IQR) 5–15) d in surgically cured episodes (i.e., macroscopic assessment by the surgeon) and 40.5 (IQR 15–42) d in cases with resection margins in non-healthy bone ( p<0.0001 ). The results were similar for treatment duration when we analyzed the results of histological examination: 13 (IQR 8–42) d for healthy bone vs. 29 (IQR 13–42) d for confirmed osteomyelitis ( p=0.026 ).

*Associations with the number of surgical interventions*. Patients with HbA1c levels of <6.5 % (prediabetes) more often required a revision during the same hospitalization than those with HbA1c levels of ≥6.5 % did (29.4 % vs. 12.1 %, respectively, p=0.023 ). A cut-off level of ≥7.1 % HbA1c yielded similar results (26.1 % vs. 11.3 %, respectively, p=0.038 ). In addition, in patients who underwent revision surgery after amputation ( n=20 ), those with prediabetes required multiple revisions more often than those with HbA1c levels of ≥6.5 % did (60 % vs. 10 %, respectively, p=0.019 ).

## 4 Discussion

In our cohort of patients with DFI who were referred for surgery, the main findings were as follows: (1) there was a high burden of comorbidities, (2) lower HbA1c values correlated more often with revision surgery during the same hospitalization and presented a higher number of revisions, (3) there was high compliance to biopsy sampling recommendations (i.e., number of samples per intervention), and (4) the duration of ABT in surgically cured episodes was shorter than in cases with resection margins in non-healthy bone.

In nearly 65 % of the episodes, the patients presented with a Charlson Comorbidity Index score of ≥5 points, which corresponds to an estimated 10-year survival of less than 20 % (Charlson et al., 1987). Long-term survival in patients with diabetic foot disease remains poor, especially with peripheral artery disease (PAD) or renal insufficiency (Morbach et al., 2012). These two organ complications of diabetes mellitus were present in our cohort in more than a third (36.3 % and 40 %, respectively). Polyneuropathy was noted in more than half of our patients (51.3 %). Polyneuropathy often co-exists with PAD, favoring neuroischemic ulceration (Boulton et al., 2018).

A two-sided repartition of HbA1c values was seen in our cohort, with significantly higher revision rates after previous amputation for HbA1c values of <6.5 . Rubio et al. (2020) reported an association between lower HbA1c values and a worse outcome as an independent risk factor of mortality. We do not have a scientific explanation for this observation. It is possible that a lower HbA1c value leads to a less aggressive surgical procedure, with a risk of failure in the infection treatment and requiring a second intervention.

In our cohort, all patients were referred for a surgical intervention. Thus, the entire cohort is a selection of patients with DFI that requires a surgical intervention. Not surprisingly, an amputation was indicated in 86 % of patients, with a mean number of nearly 1.5 amputations per patient. PAD is an independent risk factor for amputation in patients with or without diabetes (Berli et al., 2023; Gurney et al., 2018). We found a moderate- to high-risk WIfI score (stages 3 and 4) in almost 55 % of patients. As the clinical WIfI score increases, the limb salvage rate and amputation-free survival rate decrease, with a higher major amputation rate at 1 year (Zhan et al., 2015; Cull et al., 2014).

Clinicians and surgeons often face the problem of interpreting positive culture results after toe or forefoot amputation. While the surgical intervention indicated – macroscopically – resection of all infected bone, microbiological culture still shows evidence of growth. This microbiological result may be because of contamination of samples in the infection situs by adjacent soft-tissue infection. If there is no histopathological examination, the question of osteomyelitis cannot be answered properly in these cases. Thus, the importance of obtaining biopsies for both histological and microbiological examination is essential. This has been shown previously by Mijuskovic et al. (2018) and should guide the decision process on ABT to avoid inappropriately long antibiotic regimens.

Our results showed a relatively high congruency with sampling recommendations (Table 1) at 71.8 % of culture biopsies but only 63.2 % of histopathological samplings. However, the sampling of proximal bone biopsies and exact labeling occurred in less than 50 % of samples. This leads to difficulties in interpreting the sampling results.

In their systematic review, Pratama et al. (2022) analyzed comparative studies in terms of antibiotic regimens and treatment duration in DFI. Pham et al. (2022) showed no difference in remission rates in a 10 d vs. a 20 d antibiotic regimen after surgical debridement. Other recent trials have investigated the duration of systemic ABT in non-surgically debrided DFO, reporting a sufficient duration of 3 to 6 weeks to prevent failure (Gariani et al., 2021; Tone et al., 2015).

Our results of ABT duration (40.5; IQR 15–42 d) are in accordance with guidelines that recommend 6 weeks in cases with residual osteomyelitis and less than 2 weeks (9; IQR 5–15 d) in surgically cured episodes ( p<0.0001 ). We found similar results in terms of histological analysis, with a shortening of antibiotic duration to less than 2 weeks (13; IQR 8–42 d) in healthy bone and a significantly longer duration (29; IQR 13–42 d) in confirmed osteomyelitis ( p=0.026 ). Preoperative MRI is not always available and should not delay treatment in DFI, although we noticed a significant impact on our clinical decision process.

Our study has limitations, including its retrospective character. This also limits the interpretation of treatment decision rationale. For all cases we are unable to reconstruct why a given surgical procedure was chosen. The population is a selection of patients with DFI referred to an orthopedic-infection unit. Also, although we aim for an antibiotic-free interval prior to surgery, based on this retrospective analysis, this was not possible in 41.9 % of the cases because of concomitant severe skin- and soft-tissue infection. In more complex cases, the antibiotic regimen was changed or adapted frequently. Therefore, it was impossible to collect all antibiotic agents in a standardized way for statistical analysis. Because this study focused on the postoperative duration of ABT and not the antibiotic agents used itself, this limitation had likely no influence on the outcome results. We are unable to reconstruct the reasoning for not sampling in the episodes without biopsy results. While – according to protocol – biopsy samples should not be obtained in cases of clear resection in healthy bone, this was not always described in the operation notes. The value of histology results in the decision-making for antibiotic treatment duration is important in our institution but may not be applicable to other institutions. As noted above, the results were similar when comparing macroscopic (surgical) and microscopic (histopathologic) assessment in this regard. Finally, we did not assess whether or not adherence to in-house diagnostic or therapeutic recommendations had a better outcome because this was not the purpose of the study. Cost-effectiveness of our approach and the value of a specialized institution for DFI were not evaluated in this study. As we aim to optimize our compliance with diagnostic and therapeutic principles, cost-effectiveness calculations may follow alongside these analyses.

## 5 Conclusion

Adherence to the in-house recommendations in terms of biopsy sampling was good, moderate for sending samples for histological analysis and poor for labeling the anatomic location. Adherence to ABT duration was good but can be improved by shortening treatment duration for surgically cured cases. Patients with a DFI requiring amputation demonstrated a high prevalence of comorbidities and high disease scores and frequently underwent amputation revision. Except for revision surgery, these findings were irrespective of HbA1c values.

## Data Availability

The data that support the findings of this study are available from the corresponding author Noémie Reinert upon reasonable request.

## Appendix A Abbreviations

**Table:**

ABT	antibiotic therapy
AFS	amputation-free survival
DFI	diabetic foot infection
DFO	diabetic foot osteomyelitis
IQR	interquartile range
MRI	magnetic resonance imaging
PAD	peripheral arterial disease
WIfI	Wound, Ischemia, and foot Infection
